# Identification of dietary patterns associated with elevated blood pressure among Lebanese men: A comparison of principal component analysis with reduced rank regression and partial least square methods

**DOI:** 10.1371/journal.pone.0220942

**Published:** 2019-08-16

**Authors:** Farah Naja, Laila Itani, Nahla Hwalla, Abla M. Sibai, Samer A. Kharroubi

**Affiliations:** 1 Department of Nutrition and Food Sciences, Faculty of Agricultural and Food Sciences, American University of Beirut, Beirut, Lebanon; 2 Department of Nutrition and Dietetics, Faculty of Health Sciences, Beirut Arab University Beirut, Lebanon; 3 Department of Epidemiology and Population Health, Faculty of Health Sciences, American University of Beirut, Beirut, Lebanon; Kasturba Medical College Mangalore, INDIA

## Abstract

**Background:**

To examine the associations of dietary patterns with odds of elevated Blood Pressure (BP) among Lebanese adult males using principal component analysis (PCA), and compare the results to two other data reduction methods, including reduced rank regression (RRR) and partial least-squares (PLS) regression.

**Methods:**

Data from the National Nutrition and Non-Communicable Disease Risk Factor Survey conducted in Lebanon between years 2008 and 2009 were used. Dietary intake data were collected by a 61-item food frequency questionnaire (FFQ). In addition, anthropometric and blood pressure measurements were obtained following standard techniques. For the purpose of this study, data of males older than 20 years with no history of chronic diseases were selected (n = 673). Elevated BP was indicated if the systolic blood pressure was > = 130mm Hg and/or the diastolic blood pressure > = 85 mm Hg. Dietary patterns were constructed using PCA, PLS and RRR and compared based on the performance to identify plausible patterns associated with elevated BP. For PLS and RR, the response variables were BMI, waist circumference and percent body fat. Multiple logistic regression was used to evaluate the associations between the dietary pattern scores of each method and risk of elevated BP.

**Results:**

Three dietary patterns were identified using PCA: Western, Traditional Lebanese, and Fish and alcohol. Both the Western and the Traditional Lebanese patterns were associated with higher odds of elevated BP in the study population (OR = 1.23, CI 1.03, 1.46; OR = 1.29, CI 1.09, 1.52 respectively). The comparison among the three methods for dietary patterns derivation showed that PLS and RRR derived patterns explained greater variance in the outcome (PCA: 1.2%; PLS: 14.1%; RRR: 15.36%) and were significantly associated with elevated BP, while the PCA dietary patterns were descriptive of the study population’s real dietary habits (PCA: 23.6%; PLS: 19.8%; RRR: 11.3%).

**Conclusions:**

The Western and Traditional Lebanese dietary patterns were associated with higher odds of elevated BP among Lebanese males. The findings of this study showed that, compared to PCA, the use of RRR method resulted in more significant associations with the outcome while the PCA-derived patterns were more related to the real habits in the study population.

## Background

Despite major advances in the prevention and treatment of hypertension over the past decade, it remains a significant public health challenge and a major risk factor for cardiovascular diseases, stroke, and kidney diseases. In the Global Burden of Diseases (GBD) 2015, as well as GBD 2013, elevated systolic blood pressure was found to be the most important Level 3 risk factor globally [1]. Currently, one in every two adults worldwide is affected and such prevalence is increasing dramatically among all age groups [2]. According to the report from the Eighth Joint National Committee on recommendations for the management of elevated Blood Pressure (BP), losing weight for individuals who are overweight or obese, engaging in regular physical activity, and adopting a healthy eating plan were all the lifestyle changes that could reduce the risk of hypertension [3].

The quest for a healthy eating plan to reduce the risk of elevated BP has driven many investigations, which focused traditionally on single nutrients and foods. Among the most widely studied foods/nutrients are salt, fat, calcium, magnesium, and potassium. Although, studies addressing these single nutrients or foods have undoubtedly advanced the knowledge of the etiology and prevention of hypertension and its health implications, they presented a few conceptual and methodological challenges. Most importantly, nutrients are usually consumed as part of meals and in many cases they have either synergistic or interactive metabolic actions, making it rather difficult to separate out their specific effects. In addition, results of single nutrient dietary intake analyses are often obscured by the collinearity and interaction of the various nutrients, despite rigorous and complicated statistical techniques [4–6].

In view of these challenges, the dietary pattern approach was proposed as a complementary method to study diets and their association with disease, addressing dietary intake as a pattern rather than a sum of single nutrients consumed together. Such an approach was particularly useful in studying the association of diet with diseases of multi-factorial and complex etiology such as non-communicable diseases including hypertension and elevated BP.

Various methods have been proposed to derive dietary patterns and included theoretical/ hypothesis-driven methods (*a priori*), empirical/data-driven methods (*a posteriori*), or hybrid techniques of theoretical and empirical methods [7]. Theoretical methods are based on current scientific knowledge and generally represent nutrition guidelines and recommendations shown to be protective for a particular disease [8]. Empirical methods on the other hand, do not depend on a prior definition of a healthy pattern and rather use statistical approaches to provide information about existing dietary patterns within the population [9]. A frequently used empirical method is principal component analysis (PCA). PCA, one of the major methods for deriving dietary patterns, is a data-reduction method that generates patterns based upon inter correlations between original food intake variables [10]. Dietary patterns derived using PCA tend to explain a high proportion of the variability in dietary intake and hence describe actual dietary patterns of the population, however, these patterns may be poorly related to disease risk [11]. Recently hybrid methods, using a combination of theoretical knowledge and statistical approaches to determine dietary patterns were proposed, such as reduced rank regression (RRR). This method aims to construct linear functions of foods that best explain the variations in the outcome variables (such as a disease-related nutrient, subclinical or clinical endpoints). Therefore, compared to patterns derived by PCA, those obtained using RRR tend to be more associated with the outcome, despite not being necessarily consumed together and being in some instance behaviorally irrelevant [12]. Combining the strengths of PCA and RRR is the partial least-squares (PLS) regression analysis, which strives to identify patterns that maximize the variance explained in both dietary intake and the intermediary response variables related to the health or disease outcome. Such dietary patterns may allow for directed data reduction of food intake variables groups through specific nutrients or biomarkers of interest [13].

In the case of hypertension and elevated BP, studies using the *a priori* approach focused on three main dietary patterns including The DASH (Dietary Approaches to Stop Hypertension), Nordic and Mediterranean diets [14]. A systematic review and meta-analysis of randomized controlled trials showed that these diets lowered systolic BP and diastolic BP by 4.26 mm Hg and 2.38 mm Hg, respectively [14]. On the other hand, a meta-analysis of 27 studies using a posteriori approach (mainly PCA) showed that adherence to a ‘healthy’ dietary pattern decreased the risk of hypertension by 19%, while adherence to a heavy drinking pattern led to a 62% increase in its risk [15]. Fewer studies used the RRR methods and showed significant associations among the identified patterns and the risk of hypertension [16–18]. Compared to PCA and RRR, the PLS method was seldom used in examining the association between diet and hypertension.

Lebanon, similar to many countries of the Levant, is experiencing an epidemiological transition with surging rates of cardiovascular diseases and their risk factors including elevated BP. In 2018, a national cross sectional survey showed that 29.3% of Lebanese adults are hypertensive with the highest rates observed among males [19]. Concomitant to the epidemiological transition in the country are changes in the diet and lifestyle of the population, mainly due to increased globalization and urbanization. Previous research conducted by our group showed that the traditional Lebanese diet, characterized by fruits, vegetables, whole grains and olive oil, is slowly eroding and being replaced by a ‘western’ type of diet high in fast foods, sweets, sugar sweetened beverages and fats and oils [20]. Furthermore, a higher adherence to the western type of diets was observed among males as compared to females [20].

The higher prevalence of hypertension in males coupled to their dietary practices are alarming and call for further investigations to inform policy and public health interventions. In this study, we aimed to examine the association of dietary patterns with the odds of elevated BP among Lebanese adult males, using PCA. We have also applied RRR and PLS to the same data and compared the performance of these two methods to that of PCA.

## Methodology

### Study design and data collection

Data for this study were drawn from the Nutrition and Non-Communicable Disease Risk Factor Survey conducted in Lebanon between years 2008 and 2009 and which included a nationally representative sample of 3656 individuals aged 6 years and above. Details about the design and protocol used in this survey are described elsewhere [20]. In brief, using a stratified cluster sampling technique, households (primary sampling units) were selected and individuals were approached and invited to participate. Governorates and districts in the country constituted the strata and the clusters within the sampling frame used, respectively. One adult (>18 years) and one child (between 6 and 18 years) from each household were selected from the household excluding pregnant and lactating women and subjects with mental disabilities. In case more than one eligible adult was present in the household at the time of the interview, a household roster was used to select one randomly. Compared to the Lebanese population [21], the distribution of the study sample was similar by sex and 5-year age group. The institutional Review Board has approved the protocol used in the study, and all subjects provided written consent / assent prior to participation. For the purpose of this study, data of males older than 20 years with no history of chronic diseases (including hypertension) were used (n = 673).

Data collection took place in the households and lasted for 60 minutes. During face-to face interviews with the participants, trained field workers used a multi component questionnaire to collect information about sociodemographic characteristics, lifestyle habits and dietary intake, in addition to anthropometric and blood pressure measurements. Sociodemographic characteristics include age (in years), education level, occupation, marital status and crowding index. The latter was calculated by dividing the number of household members by the number of rooms used for sleeping as the denominator. Crowding index is used as a proxy for socioeconomic status, whereby several epidemiological studies have correlated a high household crowding index with low socioeconomic status [22, 23]. The lifestyle habits considered were smoking (smoker, non-smoker-including previous smoker) and physical activity which was assessed using the Arabic short version of the IPAQ. Subjects were grouped into three categories of physical activity (low, moderate, high) corresponding to energy requirements of the various activities in which they engaged [24]. Dietary intake was examined using an Arabic food frequency questionnaire (FFQ). The food list consisted of 61 food/food groups representing commonly consumed food items and beverages in Lebanon. Participants were asked to use reference portion sizes or grams in estimating their dietary intake. The reference portion size used was one standard serving expressed in household measures (cups, spoons and plates). In this FFQ, five choices to indicate the frequency of consumption of each item were indicated (per day, per week, per month, per year or never). The reported frequency of each food item and beverage was then converted to a daily portion intake. To be used in the derivation of dietary patterns, the 61 food items were grouped into 30 food groups, based on similarities in ingredients, nutrient profile and /or culinary usage. Certain food items with unique composition (burghol (parboiled wheat) and mayonnaise) were classified individually. Food groups were expressed as portion per day.

The anthropometric measurements included weight, height and waist circumference. Standard techniques and calibrated equipment were used to obtain these measurements. Subjects were weighed to the nearest 0.1 kg in light indoor clothing and with bare feet or stockings. Using a stadiometer, height was measured without shoes and recorded to the nearest 0.5cm. Body mass index (BMI) was calculated as the ratio of weight (kilograms) to the square of height (meters). Waist circumference was measured using a plastic measuring tape, to the nearest 0.5 cm, at the midpoint between the bottom of the rib cage and above the top of the iliac crest during minimal respiration.

Blood pressure was measured using a standard mercury sphygmomanometer, after participants were seated and rested for 5 minutes. For both systolic and diastolic blood pressure, two readings were collected at 5 minutes intervals. The average of the two readings was used in this study. Elevated BP was indicated if the systolic blood pressure was > = 130mm Hg and/or the diastolic blood pressure > = 85 mm Hg [25].

### Definition of responses

To identify potential response variables for RRR and PLS that are known to be related to hypertension and are of particular interest in this population, we reviewed previously published studies and chose the body mass index (BMI), waist circumference and percentage of body fat. These responses were consistently associated with hypertension and presumed to be important in its etiology. Previous studies indicated that a higher BMI and/or waist circumference was associated with high hypertension risk [26, 27]. Additionally, an increase of percentage of body fat increased significantly the risk of hypertension in previous epidemiologic studies [28, 29]. Altogether, compared with healthy adults, those with hypertension are likely to have higher values for all three response variables.

### Statistical methods

#### Dietary analysis

Data reduction techniques using three statistical methods (RRR, PCA, and PLS) were used to derive dietary patterns out of the 30 food groups. These methods are similar according to their mathematical foundation and their technique of deriving factors. The computations of pattern scores by using the three statistical methods depend on the calculation of eigenvalues and corresponding eigenvectors of the covariance matrix of both predictors and responses, respectively. As the eigenvalue represents the proportion of variation accounted for by the corresponding score, only the first pattern scores with the largest eigenvalues are of significance. However, in order to ensure increased comparability across the three methods, the same number of selected patterns was retained from all techniques.

The PCA, PLS, and RRR analyses were all conducted in SAS (SAS Institute Inc., Cary, North Carolina). In the analysis, a dietary data file which contains the 30 food groups and the three response variables was used, as well as sociodemographic characteristics and lifestyle. The final number of extracted factors is selected by applying random-sample cross-validation and van der Voet’s test [30]. The selected factors represent the model with residuals that were not significantly larger than the model with the minimum predicted residual sum of squares (PRESS) [31]. In our application, the absolute minimum PRESS was attained with six extracted factors. However, this was not much smaller than the PRESS with three factors. Thus, a statistical model comparison test was performed in order to test whether this difference is significant. The results in comparing the cross-validated residuals from models with 6 and 3 factors revealed that the difference between the two models is not significant; therefore, the model with fewer factors i.e. the model with three factors is preferred for the analyses.

Factor loadings, which represent the correlation between the factors and food groups, of each food group on the factors were also determined. Additionally, the percentage of factor-specific and all factor variation across the three statistical methods which explain the response variables and food groups was computed.

#### Descriptive analysis and modelling

Descriptive statistics were presented to summarize the study variables of interest as counts and percentages for the categorical variables and as means and standard deviations for the continuous ones. Chi-square and independent t-tests were used to chart comparisons of categorical and continuous variables between participants with and without elevated BP. The continuous factor scores produced by PCA, PLS, and RRR analyses for all retained factors were used to assess the association of dietary pattern scores with elevated BP status. Simple logistic regression models (Model 1) were applied to evaluate the associations between the dietary pattern scores of each method and risk of elevated BP, with the latter as dependent variable. In order to evaluate the correlates of elevated BP risk, multiple logistic regression model was utilized. In this model, variables were included if they were significantly associated with the dependent variable in the univariate analysis, excluding response variables. Hence model 2 was adjusted for age. Odds ratios and their respective 95% confidence intervals were computed. All reported *p*-values were based on two-sided tests and were compared with a significance level of 5%. The Statistical Package for the Social Sciences was used for all computations.

#### Comparison of dietary pattern methods

Previously, different approaches were used in order to assess and compare dietary pattern methods [11, 32]. In the present study, PCA, PLS and RRR methods were compared mainly based on the relative loading of food groups within each dietary pattern and its association with elevated BP. The three methods were also assessed based on the magnitude of variation of each method that explained the response variables and food groups.

## Results

### Study sample characteristics

A total of 673 study participants provided dietary data. Table 1 displays the socio-demographic and lifestyle characteristics of the overall study participants including those with and without elevated BP. Overall 310 participants had an elevated BP (46%). The average age of the study participants was 32.83±9.41 years with 294 (43.8%) married and 417 (62.8%) of crowding index (≥1). Furthermore, the sample population comprised subjects from all levels of education ranging from illiterate to primary (12.5%) to university and higher education (35.1%), with 112 (16.7%) unemployed, 286 (42.6%) employed and 274 (40.8%) are owner of business. Regarding lifestyle characteristics, a considerable proportion of participants (57.1%) reported no smoking and about 87% reported a total energy intake more than 2000 Kcal. Comparable proportions of participants were active and low physical activity (47.8% vs 52.2%). Finally, the average BMI (kg/m^2^), percent body fat (%) and waist circumference (cm) of the study participants were 26.83±4.81, 24.16±6.42 and 91.93±13.35 respectively. There were significant differences between participants with and without elevated BP by age, BMI, percent body fat and waist circumference, whereby those with elevated BP were older and had higher BMI, waist circumference, and percent body fat as compared to participants without elevated BP.

**Table 1:** Socio-demographic, anthropometric and lifestyle characteristics of study participants (n = 673).

	Total sample (n = 673)	Subjects with normal BP (n = 340)	Subjects with elevated BP (n = 310)	Significance
**Socio-demographic characteristics**				
**Age (years), Mean ± SD**	32.83±9.41	**31.93± 8.46**	**33.22±9.85**	**p<0.001**
**Education level, n (%)**				
Illiterate to primary	84(12.5)	42(12.4)	38(12.3)	χ2 = 4.182, p = 0.382
Complimentary	169(25.1)	87(25.6)	77(24.8)	
Secondary	99(14.7)	43(12.6)	52(16.8)	
Technical	85(12.6)	40(11.8)	44(14.2)	
University and higher education	236(35.1)	128(37.6)	99(31.9)	
**Occupation type, n (%)**				
Unemployed	112(16.7)	59(17.4)	47(15.2)	χ2 = 0.600, p = 0.741
Employed	286(42.6)	143(42.2)	135(43.5)	
Owner of a business	274(40.8)	137(40.4)	128(41.3)	
**Marital status, n (%)**				
Single	378(56.3)	189(55.8)	181(58.4)	χ2 = 0.459, p = 0.498
Married	294(43.8)	150(44.2)	129(41.6)	
**Crowding Index, n (%)**				
<1	247(37.2)	128(37.6)	112(36.2)	χ2 = 0.136, p = 0.712
≥1	417(62.8)	212(62.4)	197(63.8)	
**Anthropometric and lifestyle characteristics**				
**Body Mass Index (BMI) (kg/m2), Mean ± SD**	**26.83±4.81**	**25.91±4.25**	**27.81±5.15**	**p = 0.009**
**Percent body fat (%), Mean ± SD**	**24.16±6.42**	**22.80±6.02**	**25.42±6.32**	**p<0.001**
**Waist circumference (cm), Mean ± SD**	**91.93±13.35**	**89.13±12.74**	**94.85±13.26**	**p<0.001**
**Total energy intake, n (%)**				
<1400 Kcal	16(2.4)	11(3.2)	5(1.6)	χ2 = 3.536, p = 0.171
1400 ≤ energy intake<2000 Kcal	74(11.0)	41(12.1)	28(9.0)	
≥2000 Kcal	583(86.6)	288(84.7)	277(89.4)	
**Physical activity, n (%)**				
Low	311(52.2)	168(55.6)	133(48.0)	χ2 = 3.356, p = 0.067
Active (including moderate and high)	285(47.8)	134(44.4)	144(52.0)	
**Smoking status, n (%)**				
No	384(57.1)	194(57.1)	177(57.3)	χ2 = 0.003, p = 0.954
Yes	288(42.9)	146(42.9)	132(42.7)	

### Dietary patterns

Using van der Voet’s criteria [30], three factors for the three statistical methods (PCA, PLS and RRR) were retained based on the minimum PRESS. The main factor loadings of the three retained patterns derived by the three methods are presented in Table 2. A high positive loading indicates a strong direct association between the food group and the pattern, whereas a high negative loading reflects a strong inverse association. The major contributors to the first PCA pattern (“Western” pattern) were fried potato, refined grains, regular soda, sweets, pizza and pies, cured meat, fast food sandwiches, bottled fruit juices, whole dairy products, meat and poultry, fats and oils, nuts and seeds and ice cream, all of which were positively correlated with the pattern score. The majority of the aforementioned food groups loaded positively on factor 2 of the RRR and PLS methods. Factor 2 (‘Traditional Lebanese” pattern), based on the PCA analyses, was characterized by high positive loadings of fruits, vegetables, legumes, olives, whole bread, hot drinks, dried fruits, burghol, starchy vegetables and eggs. In contrast, the first PLS pattern had high positive loadings of the same food groups, whilst positive loadings were scattered across with the three factors identified using RRR. Factor 3 (‘Fish and alcohol” pattern) of the PCA was characterized by high positive loadings of fish, alcoholic beverages, light soda, low fat dairy products, mayonnaise and breakfast cereals as well as by a negative loading of Turkish coffee. High positive loadings of the aforementioned food groups were scattered across all factors of the other two methods.

**Table 2:** Factor loadings of food groups in dietary patterns identified using principle component analysis, reduced-rank regression and partial least-squares (n = 673).

	Principle component analysis			Partial least-squares			Reduced-rank regression		
Food groups	Western pattern	Traditional Lebanese pattern	Fish pattern	Factor 1	Western-_PLS	Factor 3	Factor 1	Western-RRR	Factor 3
Fried potato	0.34	-0.16	-	-0.31	0.34	-	-0.20	0.25	-
Refined grains	0.31	-	-0.29	-0.28	0.27	-0.15	-0.27	0.30	0.11
Regular soda	0.30	-0.26	-	-0.19	0.33	-	-	0.08	0.19
Sweets	0.27	-	-0.19	-0.22	0.21	-0.18	-0.19	0.18	-0.30
Pizza and pies	0.25	-0.27		-	0.35	-	0.11	0.24	-
Cured meat	0.24	-0.12	0.12	-	0.28	-	-	0.23	-
Fast food sandwiches	0.21	-0.33	0.14	-0.20	0.29	0.12	-	0.20	-
Bottled fruit juices	0.21	0.11	-0.11	—	0.19	-0.22	-	0.31	-
Whole dairy products	0.21	0.20	-	0.29	0.20	-0.20	0.34	-	-
Meat and poultry	0.21	-	0.23	-	0.21	-0.12	-	0.29	-
Fats and oils	0.20	0.15	-0.21	-	0.20	-	0.15	0.11	-0.24
Nuts and seeds	0.20	-	0.16	-	0.19	-0.27	-	0.24	0.34
Ice cream	0.18	-	-	-	0.20	-0.16	-	0.33	-0.47
Fruits	0.16	0.34	-	-	-	-0.46	-0.12	-	-0.33
Vegetables	0.16	0.34	0.20	0.34	0.10	-0.28	0.36	-	-
Legumes	0.16	0.24	-	0.10	0.11	-0.19	0.14	-	-
Olives	0.18	0.22	-	—	0.13	-0.25	-	0.24	-0.13
Whole bread	-0.10	0.20	0.29	0.32	-	-0.14	0.25	0.12	-
Hot drinks	-	0.19	-	-	-	-	-	-0.13	0.11
Dried fruits	-	0.18	-	-	-	-0.21	-	-	-0.12
Burghol (crushed wheat)	-	0.18	-	0.13	-	-0.23	-	-0.10	-0.15
Starchy vegetables	0.10	0.14	0.14	0.21	0.10	-0.18	0.23	0.20	-
Eggs	0.13	0.13	-	-0.10	0.10	-0.16	-	0.23	0.12
Fish	0.14	0.10	0.39	-	-	-0.32	-0.12	0.13	-
Alcoholic beverages	-	-	0.34	-0.11	-	-0.10	-0.13	-	-0.13
Light soda	-	-0.11	0.28	0.33	0.14	-	0.41	0.22	-
Low fat dairy products	-	-	0.28	-	-0.10	-	-	-0.13	0.27
Mayonnaise	0.16	-0.26	0.23	-0.23	0.16	-	-0.24	0.11	-
Breakfast cereals	-	-	0.18	-0.27	-	-	-0.27	0.10	0.31
Turkish coffee	0.10	-	-0.13	0.17	0.12	-	0.25	-	-

The color gradation denotes the direction of the correlation between the food groups and the dietary patterns. Dark, mild and light green represent the food groups of the Western, Traditional Lebanese and Fish patterns respectively as derived from PCA.

Loadings lower than │0.1│ were deleted for simplicity

### Explained variations in response variables and food groups

The variations in responses and food groups by dietary patterns identified using three statistical methods (PCA, PLS and RRR) are summarized in Table 3. As expected, PCA explained the least amount of variation in response variables (1.2%), followed by PLS (14.1%) and RRR (15.36%). However, the difference in explained response variability for PLS and RRR was relatively small.

**Table 3:** Explained variation (%) in response and food groups by dietary patterns identified using principle component analysis, reduced-rank regression and partial least-squares (n = 673).

Factors	Proportion (%) of explained variation in responses												Proportion (%) of explained variation in food groups		
	Principle component analysis				Partial least-squares				Reduced-rank regression				Principle component analysis	Partial least-squares	Reduced -rank regression
	Body Mass Index (BMI) (kg/m^2^)	Percent (%) Body fat	Waist circum-ference (cm)	Total	Body Mass Index (BMI) (kg/m^2^)	Percent (%) Body fat	Waist circum-ference (cm)	Total	Body Mass Index (BMI) (kg/m^2^)	Percent (%) Body fat	Waist circum-ference (cm)	Total			
**Western pattern**	0.10	0.90	0.04	0.35	9.33	14.94	11.31	11.86	12.01	15.91	13.56	13.83	11.71	4.13	2.97
**Traditional Lebanese pattern**	0.30	1.84	0.74	0.61	11.73	14.95	13.13	1.41	13.04	18.05	13.96	1.19	6.41	9.63	5.18
**Fish pattern**	0.72	1.93	0.95	0.24	12.28	16.56	13.47	0.83	13.47	18.07	14.54	0.34	5.46	6.00	3.18
**Total**				1.20				14.10				15.36	23.59	19.76	11.33

Using PCA, 23.6% of variation in predictors (food groups) was found, compared to 19.8% of PLS and 11.3% of RRR. It is worth noting that, while the first reduced rank regression factor explains 13.8% of the response variation, it accounts for only about 3% of the predictor variation. In contrast, the first principal components regression factor accounts for most of the predictor variation (11.7%) but only 0.35% of the response variation. The first partial least squares factor balances the goals of explaining response and predictor variation with 11.9% and 4.1% respectively.

### Dietary patterns and elevated BP

Table 4 provides the different associations of factors identified by PCA, PLS and RRR with odds of elevated BP. In the crude models, whilst only one factor from each of the PCA and PLS methods was significantly associated with elevated BP (“Traditional Lebanese” pattern-PCA: OR = 1.31, CI 1.11, 1.54; “Western” pattern—-PLS: OR = 1.14, CI 1.03, 1.26), two factors from the RRR method were found to be significantly associated with elevated BP (factor 1-RRR: OR = 1.35, CI 1.21, 1.50; Western-RRR: OR = 1.28, CI 1.06, 1.53). In the multivariate adjusted models, Western-PLS pattern remained significantly associated with elevated BP (OR = 1.15, CI 1.04, 1.28). However, more dietary factors determined by the PCA and RRR methods were found to be associated with elevated BP. In particular, both the “Western” and “Traditional Lebanese” patterns from the PCA were significantly associated with higher odds of elevated BP (OR = 1.23, CI 1.03, 1.46; OR = 1.29, CI 1.09, 1.52 respectively), whereas all factors determined by the RRR were found to be significantly associated with elevated BP (factor 1: OR = 1.38, CI 1.22, 1.55; “Western” pattern—RRR: OR = 1.34, CI 1.10, 1.64; factor 3: OR = 0.64, CI 0.44, 0.94).

**Table 4:** Odds ratios and 95% confidence intervals for elevated BP and factor scores derived using principal component analysis, reduced-rank regression and partial least-squares (n = 673).

	OR (95% confidence interval)	
	Model 1	Model 2
**Principle component analysis**		
“Western” pattern	1.17(0.99,1.37)	**1.23 (1.03–1.46)**, p = 0.021
“Traditional Lebanese” pattern	**1.31(1.11,1.54), p = 0.001**	**1.29 (1.09–1.52)**, p = 0.002
“Fish and alcohol” pattern	0.94(0.81,1.10)	0.96 (0.82–1.12)
**Partial least-squares regression**		
Factor 1	1.09(0.95,1.25)	1.05 (0.91–1.22)
“Western”_PLS	**1.14(1.03,1.26), p = 0.012**	**1.15 (1.04–1.28)**, p = 0.006
Factor 3	0.91(0.81,1.02)	0.90 (080–1.02)
**Reduced-rank regression**		
Factor 1	**1.35(1.21,1.50), p < 0.001**	**1.38 (1.22–1.55), p < 0.001**
“Western”_RRR	1.28 (1.06–1.53), p = 0.009	**1.34 (1.10–1.64)**, p = 0.004
Factor 3	0.63 (0.43–0.92), p = 0.017	**0.64 (0.44–0.94)**, p = 0.023

*Model 1* is the crude model

*Model 2* was adjusted for age.

## Discussion

To our knowledge, the present study was the first to examine the associations of dietary patterns with odds of elevated BP among Lebanese adults males using PCA, and comparing the results to two other data reduction methods, including RRR and PLS. Three distinct dietary patterns were identified: Western, Traditional Lebanese and Fish and Alcohol. The Western pattern was characterized mainly by a high consumption of fried potatoes; refine grains, sweets, pizza and pies and fast-food. The traditional Lebanese pattern reflected high intakes of fruit, vegetables, legumes, olives and whole wheat bread. The fish and alcohol pattern was, as name depicts, characterized by a high consumption of fish and alcohol, in addition to light soda drinks and low fat dairy products. Similar dietary patterns to those obtained in this study were also identified among a national sample of Lebanese adults [20].

In assessing the association between dietary patterns and elevated BP, the Western dietary pattern was associated with higher odds of elevated BP among Lebanese males. Such an association could be explained by the greater intakes of energy, and foods high in saturated fats, and sugars, all of which may contribute to the elevated BP. A recent meta-analysis of 28 prospective studies showed that among various food groups, meat, processed meat and sugar sweetened beverages were associated with higher relative risk of hypertension [33]. In addition, excessive energy intake and its inevitable consequence, obesity, are postulated to be major causes of hypertension [34, 35]. This association between consumption of a Western dietary pattern and risk of hypertension has also been reported in previous studies [36, 37]. For instance, among a sample of Iranian adolescents, the adjusted means of systolic and diastolic blood pressures were significantly higher among participants in the highest tertile of the Western dietary pattern scores as compared to those in the lowest tertile [36]. Furthermore, a cross-sectional study from a representative sample of the Korean population showed that the Western pattern was associated with the prevalence of hypertension only among men [38].

Our findings showed that the Lebanese Traditional dietary pattern was also associated with higher odds of elevated BP. A previous investigation of dietary patterns and components of the metabolic syndrome among Lebanese adults showed that a higher adherence to a Lebanese Traditional dietary pattern is associated with higher odds of hypertension [39]. Although this pattern consisted of many food/food groups that are known to be protective against hypertension (fruits, vegetables and whole grains) [33], it also included the traditional Lebanese foods which are high in sodium content. More specifically, a recent study by the LASH (Lebanese Action on Sodium and Health) showed that the Lebanese bread and bread-like products and dairy products, both of which are parts of the Traditional Lebanese dietary pattern as found in this study, are the main sources of sodium in the Lebanese diet (26% and 9% respectively). Examples of traditional bread/bread like products in Lebanon that are high sources of sodium is the cheese or thyme filled Manoushe, a popular breakfast, which contains 716 mg/100 gm. Labneh (strained yogurt) a commonly consumed dairy products has 278 mg/100 g [40]. Previous research also showed that, of dietary patterns prevalent among Lebanese adolescents, the Traditional Lebanese dietary pattern had the highest energy adjusted correlation with sodium intake [41].

The findings of this study did not show any association between the Fish and alcohol pattern and the odds of elevated BP. It could be argued that, in this pattern, the postulated protective effect of fish on BP was counteracted by the deleterious effect of alcoholic beverages included in this pattern [42, 43].

The second objective of the study was to compare the results obtained by PCA to those of RRR and PLS. Three important considerations ought to be examined when evaluating the utility of various data reduction methods in nutritional epidemiology: 1) the interpretability of the derived factors/dietary patterns and 2) the strength of associations of these patterns with the main outcome of interest and 3) the amount of variance in exposures and responses that these patterns explained.

With regards to the interpretability of the patterns, the PCA-derived patterns reflected to a large extent the eating behavior of the studied population. Previous investigations among Lebanese adults, older adults, adolescents and children consistently showed the prevalence of similar Western and the Traditional Lebanese patterns [20, 39, 44, 45]. In this study, the patterns obtained by RRR and PLS, though shared some characteristics of the PCA derived patterns did not result in distinct patterns that could relate to the actual food consumption in the population. For example, the second pattern of the RRR shared many food components with the PCA Western dietary pattern however it did not include important elements of what is conventionally known as ‘Western’ diet such as the soft drinks, fats and oils or the ice cream foods/food group. Similarly, the first factor of the PLS included many foods/food groups which represent the Traditional Lebanese dietary pattern as obtained by PCA, such vegetables, whole bread, and starchy vegetables, but missed important pillars of this traditional pattern like fruits, legumes, and olives. As such, similar to previous studies, in this study the dietary patterns derived by PCA are more likely to reflect real-world dietary patterns and provide clearer understanding of dietary patterns within the target population. This advantage of the PCA allowed for the formulation of tailored and context-sensitive nutrition interventions [37, 46, 47].

A main criticism of the PCA, however, is that the behavior-related patterns obtained by this method do not necessarily predict the disease of interest [48]. This argument was supported by the findings of this study, whereby-after adjustment for potential confounders- all of the RRR-derived patterns were significantly associated with the odds of elevated BP, while two PCA and only one PLS derived patterns showed significant associations with elevated BP. In accordance with our findings, other studies also showed that dietary patterns obtained by using the RRR method had stronger association with diseases than those derived by PCA or PLS [46, 49, 50]. These findings could be explained by the fact that the patterns derived from RRR and PLS are driven from disease-associated responses while the factors obtained from PCA are more reflective of the dietary habits of the population (that is, food groups consumed together in a particular population) [51]. In fact, in our study, RRR and PLS derived patterns explained the most in percent variation of responses, with the least percent observed belonging to the PCA while the patterns derived from PCA had the highest percentage compared to PLS and RRR, which had similar values.

In addition, our findings may help in clarifying the most appropriate context in which to apply each of the three methods. Should the aim of a particular study is to test hypotheses limited to a group of predetermined response variables then RRR could be the most appropriate method. On the other hand, if the aim is exploratory of diet-disease association then PCA could be the appropriate choice for deriving the patterns. PLS strikes a compromise between PCA and RRR. In this particular study, our aim was to examine the associations between relevant dietary exposures among Lebanese adult males and elevated BP risk. Hence, through applying the three methods, knowledge about important elevated BP-related dietary exposures, which were not explicitly used as response variables, was gained. PLS comprises information about mediator variables on the pathway to disease in deriving the dietary patterns and gives extra flexibility than RRR, which may lead to the detection of important disease-related dietary exposures in a population. Our results showed that PCA was the most flexible of the three methods applied here, thus allowing for the identification of the maximum number of patterns related to elevated BP, including the two patterns associated with increased risk of elevated BP. A main criticism of the PCA is being a purely data-driven method, however, it may have advantages over the other two methods (PLS and RRR) should a more exploratory analysis is needed.

The findings of this study ought to be considered in light of a few limitations. The cross sectional nature of the study limited any inference regarding causality inference between dietary patterns and elevated BP. In order to decrease the effect of reverse causality, individuals with chronic diseases (including hypertension) were excluded from the analysis. In addition, the self-reported nature of dietary intake is prone to measurement and recalls errors, despite the research team’s efforts to train the data collectors in order to standardize data collection techniques and minimize such errors. Furthermore, although the FFQ used in this study was not validated in the study population, it has been previously used among Lebanese adults and has yielded plausible results especially in relation to obesity and several metabolic abnormalities [20, 52, 53]. With regards to the dietary patterns analyses, limitations arose from a few subjective decisions that were undertaken, namely the food groupings, the number of factors to retain, and the labelling of the factors. With regard to the RRR method of deriving dietary patterns, the selection of BMI, waist circumference and percent body fat as response variables may have excluded other pathways between diet and diseases [9]. Finally, PLS and RRR may result in chance associations with response variables that will not go beyond the data set used to derive dietary patterns. However, this risk was minimized by using cross-validation in order to select the dietary patterns to investigate.

In light of the aforementioned comparison of methods, the findings of this study showed that, although the use of RRR method yielded more significant association with the outcome, the PCA-derived patterns were more interpretable, related to the real habits in the study population and also yielded plausible and translatable results in terms of the associations between the patterns and elevated BP.

## Conclusion

The findings of this study showed that the three dietary patterns identified using PCA among Lebanese males were: Western, Traditional Lebanese and Fish and Alcohol patterns. Both the Western and the Traditional Lebanese patterns were associated with higher odds of elevated BP in the study population. These findings are important in light of the escalating burden of hypertension and its associated cardiovascular diseases among males in the country. Regarding the comparison of methods to derive dietary patterns, RRR and PLS derived patterns explained greater variance in the outcome and were significantly associated with elevated BP while the PCA dietary patterns were descriptive of the study population’s real dietary habits. It remains important to note that the hypotheses and objectives of a particular study must direct the selection of the statistical technique that will be used to identify dietary patterns. More specifically, should the aim of a study be exploratory of diet-disease association then PCA could be the appropriate choice for deriving the patterns. On the other hand, if a specific biological/physiological pathway between diet and diseases was to be examined then RRR and PLS would be recommended. This needs to be further investigated in future studies in different population groups, response variables and disease outcomes.

## Supporting information

S1 File Socioeconomic and Dietary Determinants of Obesity in Lebanon Household and adult Questionnaire (English version). (DOC)

S2 File Socioeconomic and Dietary Determinants of Obesity in Lebanon Household and adult Questionnaire (Arabic version). (DOC)

S3 File Data of the study. (XLSX)

## Abbreviations

BMI: Body mass index
BP: Blood Pressure
CI: Confidence Interval
DASH: Dietary Approaches to Stop Hypertension
FFQ: Food Frequency Questionnaire
GBD: Global Burden of Diseases
IPAC: International Physical Activity Questionnaire
OR: Odds Ratio
PCA: Principal Component Analysis
PLS: Partial Least-Squares
PRESS: minimum predicted residual sum of squares
RRR: Reduced Rank Regression
