# Supplementary material for: Identification of dietary patterns associated with elevated blood pressure among Lebanese men: A comparison of principal component analysis with reduced rank regression and partial least square methods
Source: PLoS One. 2019 Aug 16;14(8):e0220942. doi: 10.1371/journal.pone.0220942 (PMC6697315; doi:10.1371/journal.pone.0220942)
Supplement: S1 File — (DOC) [file pone.0220942.s001.doc]

**Socioeconomic and Dietary Determinants of Obesity in Lebanon**

**Household and adult Questionnaire**

| Family Number | -------------------------- |
| --- | --- |

|  | **1^st^ Visit** | **2^nd^ Visit** | **Last Visit** |
| --- | --- | --- | --- |
| **Date:** |  |  |  |
| **Result:** |  |  | Total Number----- |

Name of the interviewer:--------------------------------------------------- Signature:------------------

**HOUSEHOLD INFORMATION:**

a- How many rooms are there in your house other than the kitchen, the bathroom, the parking, the open air balcony?

1) Number: -----------------------

2) No answer

**Family members**

b- Who is the head of the family? ---------------------------------------------------------

c Total Family members number who usually sleep in this house (including married and those traveling): ------------------------------ (number)

|  | HR_NAM | HR_RELHH | HR_SEX | HR_AGE | HR_EDU | HR_OCC | HR_MS | Cell |
| --- | --- | --- | --- | --- | --- | --- | --- | --- |
|  | Name | **Relation with to head of family**  1. Head of family  2. Husband/Wife  3. Son/ daughter  4. Son’s wife/ Daughter’s husband  5. Grandson/ Granddaughter  6. Father/ Mother  7. father / mother in law  8. Brother/ Sister  9. Sister/ brother in law  10. Grandmother/ Grandfather  11. Nephew/ niece  12. Other relatives  13. Others/ Not relatives | **Gender**  1. Male  2. Female | **Age**  Write 0 for children younger than 1 year old | **Level of education**  1. Illiterate  2. Reads & Writes  3. Elementary school  4. Middle School  5-.High School  6.Baccalaureat 7. Higher Education 8. Doesn’t know  9. Refuses to answer | **Occupation**  0. Doesn’t work  1. Governmental employee  2. Nongovernmental employee  3. Self-employed  4. Voluntary worker (no paycheck)  5. Student  6. Retired  7. Unemployed (able to work)  8. Unemployed (unable to work)  9. Refuses to answer | **Marital Status**  1. Single  2. Married  3. Divorced  4. Separated  5. Widowed  6. Refuses to answer | **Does he have a cell**  1. No  2. Yes |
| 1 |  |  |  |  |  |  |  |  |
| 2 |  |  |  |  |  |  |  |  |
| 3 |  |  |  |  |  |  |  |  |
| 4 |  |  |  |  |  |  |  |  |
| 5 |  |  |  |  |  |  |  |  |
| 6 |  |  |  |  |  |  |  |  |
| 7 |  |  |  |  |  |  |  |  |
| 8 |  |  |  |  |  |  |  |  |
| 9 |  |  |  |  |  |  |  |  |
| 10 |  |  |  |  |  |  |  |  |
| 11 |  |  |  |  |  |  |  |  |

**This table is for choosing the person (s) involved in this study:**

| **Number of members in the family having 5 years old and more** | | | | | | | | | | | | | | | |
| --- | --- | --- | --- | --- | --- | --- | --- | --- | --- | --- | --- | --- | --- | --- | --- |
| **The first number of the family in the** | **1** | **2** | **3** | **4** | **5** | **6** | **7** | **8** | **9** | **10** | **11** | **12** | **13** | **14** | **15** |
| **0** | 1 | 1 | 1 | 1 | 3 | 3 | 2 | 8 | 3 | 9 | 3 | 11 | 12 | 3 | 5 |
| **1** | 1 | 1 | 2 | 1 | 4 | 2 | 1 | 2 | 5 | 1 | 8 | 4 | 1 | 14 | 7 |
| **2** | 1 | 1 | 3 | 4 | 1 | 1 | 4 | 5 | 5 | 3 | 6 | 1 | 2 | 9 | 11 |
| **3** | 1 | 2 | 2 | 2 | 2 | 5 | 7 | 3 | 7 | 5 | 9 | 2 | 3 | 7 | 15 |
| **4** | 1 | 1 | 1 | 3 | 1 | 6 | 1 | 6 | 1 | 10 | 8 | 9 | 7 | 11 | 1 |
| **5** | 1 | 1 | 2 | 2 | 4 | 5 | 1 | 1 | 9 | 6 | 6 | 1 | 5 | 1 | 4 |
| **6** | 1 | 1 | 2 | 4 | 2 | 4 | 4 | 7 | 9 | 2 | 8 | 6 | 4 | 10 | 13 |
| **7** | 1 | 1 | 2 | 4 | 2 | 1 | 1 | 5 | 6 | 6 | 5 | 12 | 9 | 5 | 11 |
| **8** | 1 | 2 | 2 | 4 | 1 | 4 | 4 | 6 | 7 | 6 | 9 | 4 | 8 | 2 | 12 |
| **9** | 1 | 2 | 3 | 2 | 1 | 3 | 4 | 8 | 7 | 10 | 3 | 6 | 4 | 6 | 7 |

**For the interviewer:**

In the first column, circle the number corresponding to the first number of the family; for example, if the number of the family is 5011, then circle the number 1.

In the first row, circle the number of family members, for example 7.

The point of intersection is the number of person asked for the research; for example7 and 5=>1. If the person is not present, you can choose the number that is below.

**Name of the person involved in the study (18 +): ----------------------------------------------**

**Name of the person involved in the study (< 18): ----------------------------------------------**

**ADULT QUESTIONNAIRE (18+)**

**Family Number**---------------------------------

**Individual Number**------------------------------

**1. PERSONAL INFORMATION**

1.0- Name of the person: ----------------------------------------

1.1- Sex: 1) Male

2) Female

1.2- Date of Birth (Day/Month/Year): -------------------------

1.3- Age in years: ---------------------

1.4- Educational Level; what is the highest educational level that you got?

1) Illiterate 6) Technical Diploma

2) Reads and Writes 7) Bachelor

3) Elementary School 8) Higher education ( MSc., PhD)

4) Middle school 9) don’t know

5) High School 10) Refused to answer

1.5- Marital Status:

1) Single 4) Separated

2) Married 5) Widowed

3) Divorced 6) Refused to answer

1.6- Occupation:

1) Not Working (housewife) 6) Student

2) Governmental employee 7) Retired

3) Nongovernmental employee 8) Unemployed (can work)

4) Self- employed 9) Unemployed (can’t work)

5) Volunteer worker 10) Refuse to answer

**2. SMOKING:**

2.1- Do you smoke or used to smoke cigarettes?

1) No, I never smoked

2) Yes, I currently do

3) I used to smoke and I stopped

4) Refuses to answer

**4. DIETARY HABITS AND PERCEPTIONS**

4.1- Do you usually eat breakfast?

1. Never
2. Sometimes: times per week -------------------
3. Regularly every day

4.2- Do you eat lunch?

1. Never
2. Sometimes: times per weeks --------------------
3. Regularly every day

4.3- Do you eat dinner?

1. Never
2. Sometimes: times per weeks -----------------------
3. Regularly every day

4.4- Do you eat small snacks (chips, chocolate, fruits…) between main meals?

1. Never (go to question “f”)
2. Sometimes: times per weeks ------------------------
3. Regularly every day

4.5- If the answer is “Yes”, how many snacks do you eat daily?

1. 1
2. 2
3. 3
4. 4
5. More than 4 times, specify --------------------

4.6- Do you eat while watching Television?

1. I don’t watch Television
2. Never
3. Sometimes, days per week______
4. Most of the times

4.7- In average, how many times per week will you eat outside your house?

1. Never
2. 1
3. 2
4. 3
5. More than 3 times, specify ----------------------

### 5. PHYSICAL ACTIVITY (IPAQ)

We are interested in finding out about the kinds of physical activities that people do as part of their everyday lives. The questions will ask you about the time you spent being physically active in the **last 7 days**. Please answer each question even if you do not consider yourself to be an active person. Please think about the activities you do at work, as part of your house and yard work, to get from place to place, and in your spare time for recreation, exercise or sport.

Think about all the **vigorous** activities that you did in the **last 7 days**. **Vigorous** activities refer to activities that take hard physical effort and make you breathe much harder than normal. Think only about those physical activities that you did for at least 10 minutes at a time.

5.1- During the last 7 days, on how many days did you do vigorous physical activities like heavy lifting, digging, aerobics, or fast bicycling?

1. Number of days: ------------------------------
2. No vigorous activities

- *If your answer was “No Vigorous activities”, then skip to question 5.3.*

5.2- How much time did you usually spend doing vigorous physical activities on one of those days?

1. Hours per day: --------------------------------
2. Minutes per day: -----------------------------
3. Don’t know/Not sure

Think about all the **moderate** activities that you did in the **last 7 days**. **Moderate** activities refer to activities that take moderate physical effort and make you breathe somewhat harder than normal. Think only about those physical activities that you did for at least 10 minutes at a time.

5.3- During the last 7 days, on how many days did you do moderate physical activities like carrying light loads, bicycling at a regular pace, or doubles tennis? Do not include walking.

1. Days per week: ----------------------------
2. No moderate physical activities

- *If your answer was “No moderate physical activity”, then skip to question 5.5.*

5.4- How much time did you usually spend doing moderate physical activities on one of those days?

1. Hours per day: -----------------------------
2. Minutes per day: --------------------------
3. Don’t know/not sure

Think about the time you spent **walking** in the **last 7 days**. This includes at work and at home, walking to travel from place to place, and any other walking that you might do solely for recreation, sport, exercise, or leisure.

5.5- During the last 7 days, on how many days did you walk for at least 10 minutes at a time?

1. Days per week: -------------------------
2. No walking

- *If the answer of your questions was “No walking”, the skip to question 5.7.*

5.6- How much time did you usually spend walking on one of those days?

1. Hours per day: --------------------------
2. Minutes per day: --------------------------
3. Don’t know/not sure

Think about the time you spent **sitting** on weekdays during the **last 7 days**. Include time spent at work, at home, while doing course work and during leisure time. This may include time spent sitting at a desk, visiting friends, reading, or sitting or lying down to watch television.

5.7- During the last 7 days, how much time did you spend sitting on a week day?

1. Hours per day: ---------------------
2. Minutes per day: -----------------------
3. Don’t know/ not sure

**6. HEALTH STATUS:**

6.1- Were ever told by a health care provider that you have hypertension or any other chronic health condition?

1) Yes, specify-------

2) No

**- FOOD FREQUENCY QUESTIONNAIRE**

**Think about your eating patterns during the past year while answering this questionnaire. Please indicate your usual intake of each of the following food items per Day, Week, or Month.**

**For example: Apple. If you consume 3 apples daily, write 3 in the “Day” column, if you think you average 3 apples a week over the year, write 3 in the “Week” column. However, if you rarely consume a food, let’s say once or twice a year, then tick below “Rarely/Never”.**

**Please be precise as much as you can.**

**Remember! The accuracy of the study results depends on the accuracy of your answers.**

| **Food item** | **Serving size** | **Day** | **Week** | **Month** | **Rarely / Never** |
| --- | --- | --- | --- | --- | --- |
| **Example: Apple** | 1 item |  | 3 |  |  |
| **Bread and Cereals** |  |  |  |  |  |
| 1. White bread (1 slice) | 1 slice (30g) |  |  |  |  |
| 1. Brown or whole wheat bread | 1 slice |  |  |  |  |
| 1. Bread, markouk | ¼ loaf (30g) |  |  |  |  |
| 1. Breakfast cereals, regular/ bran | 1 cup |  |  |  |  |
| 1. Sugar coated cereals | 1 cup |  |  |  |  |
| 1. Rice, white, cooked | 1 cup |  |  |  |  |
| 1. Pasta, plain, cooked | 1 cup |  |  |  |  |
| 1. Wheat, whole, cooked / Bulgur | 1 cup |  |  |  |  |
| 1. Popcorn | 1 cup |  |  |  |  |
| **Dairy products** |  |  |  |  |  |
| 1. Low-fat milk (2% fat) | 1 cup |  |  |  |  |
| 1. Whole fat milk | 1 cup |  |  |  |  |
| 1. Milk, condensed and sweetened | 1 cup |  |  |  |  |
| 1. Fat free / low fat yogurt | 1 cup |  |  |  |  |
| 1. Whole fat yogurt | 1 cup |  |  |  |  |
| 1. Cheese regular yellow ( Kashkawan, cheddar,etc..) | 1 slice (30g) |  |  |  |  |
| 1. Cheese low fat yellow ( Kashkawan, cheddar,etc..) | 1 slice (30g) |  |  |  |  |
| 1. Cheese white ( Akkawi, feta,etc..) | 1 slice (30g) |  |  |  |  |
| 1. Cheese white low fat ( Akkawi, feta,etc..) | 1 slice (30g) |  |  |  |  |
| 1. Cheese spread ( picon, Kraft,etc.) | 1 slice (30g) |  |  |  |  |
| 1. Cheese spread low fat | 1 slice (30g) |  |  |  |  |
| 1. Labneh | 2 Tbsp |  |  |  |  |
| **Fruits & Juices** |  |  |  |  |  |
| 1. Apple, fresh, small | 1 item |  |  |  |  |
| 1. Banana, medium | 1 item |  |  |  |  |
| 1. Orange (1 item) / Grapefruit (1/2 item) | 1 serving |  |  |  |  |
| 1. Kiwi | 1 small |  |  |  |  |
| 1. Mango | 1 item |  |  |  |  |
| 1. Grapes, fresh | 1 cup |  |  |  |  |
| 1. Dried fruits: raisins (2 Tbsp), dates (2), apricots (4) | 1 serving |  |  |  |  |
| 1. Canned fruits | 1 cup |  |  |  |  |
| 1. Fresh fruit juice | 1 cup |  |  |  |  |
| 1. Fresh vegetable juice: tomato / other vegetables | 1 cup |  |  |  |  |
| 1. Fruit drinks: canned/bottled | 1 cup |  |  |  |  |
| **Food item** | **Serving size** | **Day** | **Week** | **Month** | **Rarely / Never** |
| **Vegetables** |  |  |  |  |  |
| 1. Salad – green: lettuce, celery, green peppers, onions | 1 cup |  |  |  |  |
| 1. Dark green vegetables (e.g.: spinach, silq, hindbeh,…) | 1 cup |  |  |  |  |
| 1. Carrots, raw or cooked | 1 cup |  |  |  |  |
| 1. Tomatoes, fresh, medium | 1 item |  |  |  |  |
| 1. Cucumber | 1 cup |  |  |  |  |
| 1. Corn / green peas, cooked | 1 cup |  |  |  |  |
| 1. White potato, baked / boiled / mashed | 1 item |  |  |  |  |
| 1. Squash, summer (kussa), Eggplant /cooked | 1 cup |  |  |  |  |
| 1. Cauliflower/ Cabbage/ broccoli | 1 cup |  |  |  |  |
| **Meat & Alternates** |  |  |  |  |  |
| 1. Legumes: lentils, broad beans, chickpeas, etc., cooked | 1 cup |  |  |  |  |
| 1. Nuts and seeds: peanuts, almonds, sunflower seeds, etc. | 1 cup |  |  |  |  |
| 1. Beef | 1 item (90gr.) |  |  |  |  |
| 1. Lamb | 1 item (90gr.) |  |  |  |  |
| 1. Chicken | 1 item (90gr.) |  |  |  |  |
| 1. Fish, canned with oil: tuna, sardines | 1 serving (90gr.) |  |  |  |  |
| 1. Tuna canned with water | 1 serving (90gr.) |  |  |  |  |
| 1. Shellfish: shrimp, lobster, clams | 1 cup |  |  |  |  |
| 1. Fish, fresh | 1 serving (90gr.) |  |  |  |  |
| 1. Eggs, whole, large | 1 item |  |  |  |  |
| 1. Organ Meats( Liver, kidneys, brain) | 1 cup |  |  |  |  |
| 1. Luncheon meats: Bologna, salami, etc. | 1 slice (20g) |  |  |  |  |
| 1. Sausages, makanek, hot dogs | 1 item (30g) |  |  |  |  |
| **Fats and oils** |  |  |  |  |  |
| 1. Vegetable oil: corn / sunflower / soy | 1 Tbsp |  |  |  |  |
| 1. Olive oil | 1 Tbsp |  |  |  |  |
| 1. Olives | 1 item |  |  |  |  |
| 1. Vegetable ghee | 1 Tbsp |  |  |  |  |
| 1. Butter | 1 Tbsp |  |  |  |  |
| 1. Mayonnaise | 1 Tbsp |  |  |  |  |
| 1. Lard / animal ghee | 1 cup |  |  |  |  |
| **Sweets &Desserts** |  |  |  |  |  |
| 1. Cookies: chocolate chips, oatmeal, peanut butter, etc. | 1 small item |  |  |  |  |
| 1. Doughnut / muffin | 1 item |  |  |  |  |
| 1. Cake | 1 item |  |  |  |  |
| 1. Pudding or custard, regular | 1 cup |  |  |  |  |
| 1. Ice cream | 1 cup |  |  |  |  |
| 1. Chocolate bar | 1 item |  |  |  |  |
| 1. Sugar, | 1 Tbsp |  |  |  |  |
| 1. Halawa | 1 potion (40g) |  |  |  |  |
| 1. Molasses , honey, jam | 1 Tbsp |  |  |  |  |
| 1. Arabic sweets, baklawa, maamoul, Knefeh | 1 item (40g) |  |  |  |  |

| **Food item** | **Serving size** | **Day** | **Week** | **Month** | **Rarely / Never** |
| --- | --- | --- | --- | --- | --- |
| **Beverages** |  |  |  |  |  |
| 1. Soft drinks, regular (1 can = 1½ cup) | 1½ cup (11 fl. oz) |  |  |  |  |
| 1. Soft drinks, diet (1 can = 1½ cup) | 1½ cup (11 fl. oz) |  |  |  |  |
| 1. Turkish coffee (1 small cup = ¼ cup) | ¼ cup (2 fl oz) |  |  |  |  |
| 1. Coffee/Nescafe or tea | 1 cup |  |  |  |  |
| 1. Coffee or tea, decaffeinated | 1 cup |  |  |  |  |
| 1. Hot chocolate or cocoa | 1 cup |  |  |  |  |
| 1. Beer, regular (1 can = 1½ cup) | 1½ cup |  |  |  |  |
| 1. Wine: red, white, or blush | ½ cup (4 fl. oz) |  |  |  |  |
| 1. Liquor: whiskey, vodka, gin, rum | 1/6 cup (1.5 fl oz.) |  |  |  |  |
| **Miscellaneous** |  |  |  |  |  |
| 1. Manaeesh, zaatar, cheese | 1 large |  |  |  |  |
| 1. French fries | 1 cup |  |  |  |  |
| 1. Chips: potato, corn, tortilla | 1 cup |  |  |  |  |
| 1. Falafel sandwich, medium | 1 item |  |  |  |  |
| 1. Chawarma sandwich, medium | 1 item |  |  |  |  |
| 1. Burgers( Beef, chicken, fish) | 1 item |  |  |  |  |
| 1. Burgers with cheese | 1 item |  |  |  |  |
| 1. pizza | 2 slices |  |  |  |  |
| 1. Croissant (plain, cheese, zaatar, chocolat) | 1 item |  |  |  |  |

**Are there any other foods not mentioned above that you usually eat at least once per week?**

*Example: pâté, cream sauce, fava beans, etc (do not include dry spices). Do not list foods that have been listed in the previous section.*

| **Other foods that you usually eat at least once /week** | **Usual serving size** | **Servings/week** |
| --- | --- | --- |
|  |  |  |
|  |  |  |
|  |  |  |
|  |  |  |
|  |  |  |

**11- ANTHROPOMETRIC MEASUREMENTS:**

| **MEASUREMENTS** | | |
| --- | --- | --- |
|  | **No.1** | **No.2** |
| **Height (Cm)** |  |  |
| **Weight (Kg)** |  |  |
| **Hip Circumference (Cm)** |  |  |
| **Waist Circumference (Cm)** |  |  |
| **Triceps (mm)** |  |  |
| **Sub scapular (mm)** |  |  |
| **Biceps (mm)** |  |  |
| **Suprailiac (mm)** |  |  |
