# Supplementary material for: Identification of dietary patterns associated with elevated blood pressure among Lebanese men: A comparison of principal component analysis with reduced rank regression and partial least square methods
Source: PLoS One. 2019 Aug 16;14(8):e0220942. doi: 10.1371/journal.pone.0220942 (PMC6697315; doi:10.1371/journal.pone.0220942)
Supplement: S2 File — (DOC) [file pone.0220942.s002.doc]

**استبيان مسح سلوكيات وعوامل الخطورة**

**للأمراض غير السارية في لبنان– \092008**

**إستمارة العائلة**

| FAM_NB | **رقم العائلة** | |  |  |  |  | | --- | --- | --- | --- | |
| --- | --- | --- | --- | --- | --- | --- |

|  | **الزيارة الاولى** | **الزيارة الثانية و النهائية** |
| --- | --- | --- |
| **التاريخ :** | - **- -- - - - - - - ----** | **- - - - - - - -- - ------** |
| **نتيجة الراشد** | 1. **إكتملت** 2. **مقابلة جزئية** 3. **رفض** 4. **ليس موجود في البيت** | 1. **إكتملت** 2. **مقابلة جزئية** 3. **رفض** 4. **ليس موجود في البيت** |
| **نتيجة القاصر** | 1. **إكتملت** 2. **مقابلة جزئية** 3. **رفض** 4. **ليس موجود في البيت** | 1. **إكتملت** 2. **مقابلة جزئية** 3. **رفض** 4. **ليس موجود في البيت** |

**اسم الباحث الميداني : ............................................. توقيعه :..........................................................**

| **أفراد الأسرة** | | |
| --- | --- | --- |
|  |  |  |
| TOT_FAM | **- ما هو عدد الأشخاص المقيمين في هذا البيت ما عدا الخدم؟** | ) --------------------------عدد) |
|  | **- ما هو عدد الغرف في هذا البيت ما عدا المطبخ الحمام والشرفات؟** | ) --------------------------عدد) |

| **أفراد الأسرة HR : عدد الأشخاص المقيمون حاليا في هذا المنزل حسب الجدول التالي ( لا تدخل الخدم)** | | | | | | | | |
| --- | --- | --- | --- | --- | --- | --- | --- | --- |
|  | **HR_NAM** | **HR_RELHH** | **HR_SEX** | **HR_AGE** | **HR_EDU** | **HR_OCC** | **HR_MS** | **CELL** |
|  | **الاسم** | **العلاقة مع رب الاسرة**   1. رب الأسرة 2. زوج \ زوجة 3. إبن \ إبنة 4. زوجة الإبن\ زوج الإبنة 5. حفيد\ حفيدة 6. أب \ أم 7. أب \ أم القرين 8. أخ\ أخت 9. اخ \ أخت القرين 10. جد \ جدة 11. ابن اخ\ ابن أخت 12. أقارب آخرون 13. آخرون غير أقارب | **الجنس**  1- ذكر  -2 أنثى | **العمر**  (دوّن صفر للأطفال الذين تقل أعمارهم عن سنة) | **مستوى التعليم**   1. أمي 2. يقرأ و يكتب 3. المدرسة الابتدائية 4. متوسط 5. المرحلة الثانوية 6. دبلوم مهنى 7. جامعي ( بكالوريوس ) 8. شهادة عليا ( الماجستير والدكتوراه )     77- لا يعرف  -88رفض الإجابة | **المهنة**  0- لا يعمل  1- موظف حكومي  -2موظف في القطاع الخاص  -3صاحب مصلحة او مؤسسة خاصة  -4عمل تطوعي بدون اجر  -5طالب  -6متقاعد  -7عاطل عن العمل – ولكن يبحث عن عمل  -8عاطل عن العمل – غير قادر على العمل  77- لا يعرف  -88رفض الإجابة | **الحالة الإجتماعية**  1- أعزب  -2متزوج  -3مطلق  -4منفصل  -5أرمل  -88رفض الإجابة | **لديه خليوي**  0-لا  1- نعم  77- لا يعرف  -88رفض الإجابة |
| 1 | رب الأسرة: | **1** |  |  |  |  |  |  |
| 2 |  |  |  |  |  |  |  |  |
| 3 |  |  |  |  |  |  |  |  |
| 4 |  |  |  |  |  |  |  |  |
| 5 |  |  |  |  |  |  |  |  |
| 6 |  |  |  |  |  |  |  |  |
| 7 |  |  |  |  |  |  |  |  |
| 8 |  |  |  |  |  |  |  |  |
| 9 |  |  |  |  |  |  |  |  |
| 10 |  |  |  |  |  |  |  |  |
| 11 |  |  |  |  |  |  |  |  |
| 12 |  |  |  |  |  |  |  |  |
| 13 |  |  |  |  |  |  |  |  |
| 14 |  |  |  |  |  |  |  |  |
| 15 |  |  |  |  |  |  |  |  |

**هذا الجدول لأختيار الشخص (أو الأشخاص) المشمول في الدراسة**

| **عدد أفراد الأسرة** | | | | | | | | | | | | | | | |
| --- | --- | --- | --- | --- | --- | --- | --- | --- | --- | --- | --- | --- | --- | --- | --- |
| **الرقم الأول للأسرة في العينة** | **1** | **2** | **3** | **4** | **5** | **6** | **7** | **8** | **9** | **10** | **11** | **12** | **13** | **14** | **15** |
| **0** | 1 | 1 | 1 | 1 | 3 | 3 | 2 | 4 | 3 | 9 | 3 | 11 | 12 | 3 | 5 |
| **1** | 1 | 1 | 2 | 1 | 4 | 2 | 1 | 2 | 5 | 1 | 8 | 4 | 1 | 14 | 7 |
| **2** | 1 | 1 | 3 | 4 | 1 | 1 | 4 | 5 | **1** | 3 | 6 | 1 | 2 | 9 | 11 |
| **3** | 1 | 2 | **1** | 2 | **1** | 5 | **1** | 3 | 7 | 5 | 9 | 2 | 3 | 7 | **1** |
| **4** | 1 | 1 | 1 | 3 | 1 | 6 | 1 | 6 | 1 | 10 | **1** | 9 | 7 | 11 | 1 |
| **5** | 1 | 1 | **1** | 2 | 4 | 5 | 1 | 1 | **4** | 6 | 6 | 1 | 5 | 1 | 4 |
| **6** | 1 | 1 | 2 | **1** | 2 | 4 | **6** | 7 | 9 | **1** | **7** | 6 | 4 | 10 | 13 |
| **7** | 1 | 1 | **1** | **3** | **5** | 1 | 1 | 5 | 6 | **4** | 5 | 12 | 9 | 5 | 11 |
| **8** | 1 | 2 | 2 | 4 | 1 | 4 | **5** | 6 | **1** | 6 | 9 | 4 | 8 | **1** | 12 |
| **9** | 1 | 2 | 3 | **1** | 1 | 3 | **3** | **1** | 7 | **1** | 3 | 6 | 4 | 6 | 7 |

**اسم ورقم الشخص الراشد المشمول في الدراسة--------------------------------------**

**اسم ورقم الشخص القاصر المشمول في الدراسة--------------------------------------**

**الى الباحث: اذا كان الشخص المشمول أقل من 12 سنة يسأل الشخص المسؤول في المنزل أو الشخص الذي بامكانه اعطاء معلومات كافية.**

**إستمارة الراشد ( 18 سنة وما فوق)**

| FAM_NB | **رقم العائلة** | |  |  |  |  | | --- | --- | --- | --- | |
| --- | --- | --- | --- | --- | --- | --- |
| IND_NB | **رقم الفرد** | |  |  | | --- | --- | |

| **1. البـيـــانــــات الفردية** |
| --- |

|  | **الأسئلة** | | **الأستجابة** | **Code number** |
| --- | --- | --- | --- | --- |
| **1.0** | **إسم الشخص** | | ---------------------------------- | NAME |
| **1.1** | **الجنس** | | 1 -ذكر  -2 أنثى | SEX |
| **1.2** | **تاريخ الميلاد** | | يوم / شهر / سنة  ...../........./........ | DOB |
| **1.3** | **كم عمرك الآن بالسنوات** | | **----- ----- سنة** | AGE |
| **1.4** | **المستوى التعليمي :**  **ما هو أعلى مستوى تعليمي حصلت عليه بالدراسة** | | 1. أمي 2. يقرأ و يكتب 3. المدرسة الابتدائية 4. متوسط 5. المرحلة الثانوية 6. دبلوم مهنى 7. جامعي ( بكالوريوس، ليسانس ) 8. شهادة عليا ( الماجستير والدكتوراه )     -77لا يعرف  -88رفض الإجابة | EDU |
| **1.5** | **الحالة الاجتماعية :** | | -1أعزب  -2متزوج  -3مطلق  -4منفصل  -5أرمل    77- لا أعرف  -88رفض الإجابة | MS |
| **1.6** | | **العمل :**  **أي من الفئات التالية يصف عملك الاساسي خلال الأثنى عشر شهرا الماضية ؟** | 0- لا يعمل (ربة منزل)  1- موظف حكومي  -2موظف في القطاع الخاص  -3صاحب مصلحة او مؤسسة خاصة  -4عمل تطوعي بدون اجر  -5طالب  -6متقاعد  -7عاطل عن العمل - ولكن يبحث عن عمل  -8عاطل عن العمل - غير قادر على العمل    77- لا أعرف  -88رفض الإجابة | WORK |
| **1.7** | | **حدد ما هو العمل؟** | ---------------------------------------  77- لا يعمل  -88رفض الإجابة | WORK_SPC |

| **2. التدخــــــــــــين** | | | |
| --- | --- | --- | --- |
| **الرقم** | **الأسئلة** | **الأجابة** | **كود الترميز** |
| **2.1** | **هل تدخن الآن أو سبق لك أن دخنت على الأقل 100 سيجارة (5 علب دخّان) في السنوات الماضية؟** | -1 لا  2- نعم. أدخن حالياً.  3- كنت أدخن وتوقفت (دخن 100 سيجارة على الأقل في الماضي).  77- لاأعرف \ غير متأكد  -88 رفض الإجابة. | Smk |

| **4- السلوك والعادات الغذائية** | | | | | |
| --- | --- | --- | --- | --- | --- |
| **الرقم** | **الأسئلة** | **الأجابة** | | | **كود الترميز** |
| **4.1** | **عادة هل تتناول وجبة الفطور؟** | 1- ابدا | | | Breakfast |
|  |  | 2- احيانا | | كم مرة اسبوعيا:------ |  |
|  |  | 3- نعم بانتظام يوميا  -77لا أعرف \ غير متأكد  -88رفض الأجابة | | |  |
| **4.2** | **هل تتناول وجبة الغداء؟** | 1 - ابدا | | | Lunch |
|  |  | 2- احيانا | | كم مرة اسبوعيا:-------- |  |
|  |  | 3- نعم بانتظام يوميا  -77لا أعرف \ غير متأكد  -88رفض الأجابة | | |  |
| **4.3** | **هل تتناول وجبة العشاء؟** | 1 - ابدا | | | Dinner |
|  |  | 2- احيانا | | كم مرة اسبوعيا:------- |  |
|  |  | 3- نعم بانتظام يوميا  -77لا أعرف \ غير متأكد  -88رفض الأجابة | | |  |
| **4.4** | **هل تتناول وجبات صغيرة بين الوجبات الرئيسية مثل (فاكهة، شيبس، شوكولا...) ؟** | 1 – ابدا  **(إنتقل إلى سؤال 4.6)** | | | Snack |
|  |  | 2- احيانا | | كم مرة اسبوعيا:-------- |  |
|  |  | 3- نعم بانتظام يوميا  -77لا أعرف \ غير متأكد  -88رفض الأجابة | | |  |
| **4.5** | **اذا كان جوابك نعم، كم وجبة صغيرة في اليوم؟** | 1- واحدة | | | Snack_frq |
|  |  | 2-اثنين | | |  |
|  |  | 3- ثلاثة | | |  |
|  |  | 4- اربعة | | |  |
|  |  | 5- اكثر من اربعة، حدد:_______  -77لا أعرف \ غير متأكد  -88رفض الأجابة | | |  |
| **4.6** | **هل تتناول الطعام خلال مشاهدة التلفزيون؟** | 1 – ابدا | | | TV_EAT |
|  |  | 2- احيانا | كم مرة اسبوعيا:------- | |  |
|  |  | 3- نعم بانتظام يوميا  -77لا أعرف \ غير متأكد  -88رفض الأجابة | | |  |
| **4.7** | **كم مرة في الاسبوع تاكل طعام محضر خارج المنزل؟** | 0- ولا مرة | | | Eat_out |
|  |  | 1- مرة واحدة | | |  |
|  |  | 2- مرتين | | |  |
|  |  | 3- ثلاث مرات | | |  |
|  |  | 4- اكثر من ثلاث، حدد:----------  -77لا أعرف \ غير متأكد  -88رفض الأجابة | | |  |

**5- الأنشطة البدنية**

| **للباحث** والآن سوف أسألك عن الوقت الذي تمضيه بممارسة أنواع مختلفة من النشاط البدني خلال أيام الأسبوع أرجو الأجابه على هذه الأسئلة حتى لو لم تعتبر نفسك نشيطا بدنيا  **سأسألك أولا عن ألانشطة البدنية الشاقة وهي التي تسبب تعرق شديد وزيادة في ضريات القلب والتنفس .** | | | | |
| --- | --- | --- | --- | --- |
|  | **الأسئلة** | **الأجابة** | **Code number** | |
| **5.1** | **خلال ال 7 أيام الماضية كم يوم منها قمت بنشاط بدني شاق مثل : الحمل الثقيل والحفر وأعمال البناء،ركوب الدراجة السريع، الجري السريع،aerobics الخ، لمدة لا تقل عن10 دقائق متتتالية في اليوم؟** | _____ يوم في الأسبوع  -0لم أقم بأي منها  7-7لا أعرف  8-8رفض الأجابة | HEAV_WRK | |
|  | إذا كانت الأجابة لم أقم بأي منها أو لا أعرف**. إنتقل الى الأنشطة البدنية المتوسطة سؤال 5.3** | | | |
| **5.2** | **كم من الوقت تقضيه في القيام بهذا النشاط البدني الشاق في اليوم الواحد (ساعة ، دقيقة )** ؟  ملاحظة للباحث **: سجل أجابة واحدة فقط**  **أما بالدقائق أو بالساعة** | **الوقت** | HEAV_TIME_MIN  HEAV_TIME_HR | |
|  |  | دقيقة ................ |  |  |
|  |  | ساعة ..............  77- لا أعرف/ غير متأكد |  |  |
|  | | | |  |
|  | **سأسألك الأن عن الأنشطة البدنية المتوسطة وهي الأنشطة التي تسبب تعرق بسيط وزيادة قليلة في ضربات القلب والتنفس.** | | | |
| **5.3** | **خلال ال 7 أيام الماضية كم يوم منها قمت بنشاط بدني متوسط مثل حمل أشياء خفيفة ، ركوب الدراجة الخفيف او لعب الكرة الطائرة، او الهرولة ) لمدة 10 دقائق متتالية على الأقل في اليوم.**  **( لا تتضمن المشي)** | -------------يوم  -0لم أقم بأي منها    7-7لا أعرف  8-8رفض الأجابة | MOD_WRK | |
|  | اذا كانت الاجابة لم أقم بأي منها ، لا اعرف او رفض الاجابة .. **أنتقل الى الى سؤال 5.5** | | | |
| **5.4** | **كم من الوقت تقضيه في القيام بهذا النشاط البدني المتوسط في اليوم الواحد (ساعة ، دقيقة )** **؟**  ملاحظة للباحث **: سجل أجابة واحدة فقط**  **أما بالدقائق أو بالساعة** | دقيقة................ | MOD_TIME_MIN | |
|  |  | ساعة ................  77- لا أعرف / غير متأكد | MOD_TIME_HR | |

| **للباحث : سأسألك الأن عن -الأنشطة البدنية ذات العلاقة بالتنقل من والي الأماكن** **المختلفة مشيا على الأقدام مثل الذهاب الى السوبرماركت،المشي الترفيهي، أو للرياضة و للمحافظة علي اللياقة** | | | | |
| --- | --- | --- | --- | --- |
|  | **الأسئلة** | | **الأجابة** | **Code Number** |
| **5.5** | **خلال ال7 أيام الماضية كم يوم منه اذهبت مشيا على الأقدام من والى بعض الأماكن لمدة لا تقل عن 10 دقائق متواصلة على الأقل في كل مرة** | | ______يوم  -0لم أمشي  7-7لا أعرف  8-8رفض الأجابة | WALK |
| اذا كانت الاجابة لم أمشي، لا أعرف أو رفض الأجابة .... .. أنتقل الى سؤال 5.7 | | | | |
| **5.6** | **بالمتوسط كم ساعة / دقيقة تمشي في كل مرة للتنقل من والى هذة الأماكن .**  ملاحظة للباحث **: سجل أجابة واحدة فقط**  **أما بالدقائق أو بالساعة** | الوقت | |  |
|  |  | دقيقة ................ | | WALK_TIM_MIN |
|  |  | ساعة................  77- لا أعرف/ غير متأكد | | WALK_TIM_HR |

| **للباحث : سأسألك الأن عن الوقت الذي أمضيته في الجلوس في عملك، في المنزل، في المدرسة، أو في وقت الفراغ مثل زيارة الأصدقاء ، القراءة، مشاهدة التلفاز، الخ ...** | | | |
| --- | --- | --- | --- |
| **5.7** | **بالمتوسط ، خلال ال7 أيام الماضية، كم من الوقت أمضيته في الجلوس في اليوم الواحد؟(ساعة ، دقيقة )**  **؟**  ملاحظة للباحث **: سجل أجابة واحدة فقط**  **أما بالدقائق أو بالساعة** | دقيقة................ | Sedentary_MIN |
|  |  | ساعة ................  77- لا أعرف / غير متأكد | Sedentary_HR |

| **للباحث : الحالة الصحية** | | | |
| --- | --- | --- | --- |
| **6** | **هل سبق وان أخبرت من قبل مرفق صحي بان ضغط دمك مرتفع او عندك اي مرض مزمن ؟** | 1- نعم  2- لا    77- لا أعرف  88- رفض الأجابة |  |
|  |  | ساعة ................  77- لا أعرف / غير متأكد |  |

**FOOD FREQUENCY QUESTIONNAIRE**

**Think about your eating patterns during the past year while answering this questionnaire. Please indicate your usual intake of each of the following food items per Day, Week, or Month.**

**For example: Apple. If you consume 3 apples daily, write 3 in the “Day” column, if you think you average 3 apples a week over the year, write 3 in the “Week” column. However, if you rarely consume a food, let’s say once or twice a year, then tick below “Rarely/Never”.**

**Please be precise as much as you can.**

**Remember! The accuracy of the study results depends on the accuracy of your answers.**

| **Food item** | **Serving size** | **Day** | **Week** | **Month** | **Rarely / Never** |
| --- | --- | --- | --- | --- | --- |
| **Example: Apple** | 1 item |  | 3 |  |  |
| **Bread and Cereals** |  |  |  |  |  |
| 1. White bread (1 slice) | 1 slice (30g) |  |  |  |  |
| 1. Brown or whole wheat bread | 1 slice |  |  |  |  |
| 1. Breakfast cereals, regular/ bran | 1 cup |  |  |  |  |
| 1. Rice, white, cooked | 1 cup |  |  |  |  |
| 1. Pasta, plain, cooked | 1 cup |  |  |  |  |
| 1. Wheat, whole, cooked / Bulgur | 1 cup |  |  |  |  |
| **Dairy products** |  |  |  |  |  |
| 1. Low-fat milk (2% fat) | 1 cup |  |  |  |  |
| 1. Whole fat milk | 1 cup |  |  |  |  |
| 1. Fat free / low fat yogurt | 1 cup |  |  |  |  |
| 1. Whole fat yogurt | 1 cup |  |  |  |  |
| 1. Cheese regular | 1 slice (30g) |  |  |  |  |
| 1. Cheese low fat | 1 slice (30g) |  |  |  |  |
| 1. Labneh | 2 Tbsp |  |  |  |  |
| **Fruits & Juices** |  |  |  |  |  |
| 1. Citrus Orange (1 item) / Grapefruit (1/2 item) | 1 serving |  |  |  |  |
| 1. Deep Yellow or orange( Peach, plums, etc..) | 1 item |  |  |  |  |
| 1. strawberry | 1 cup |  |  |  |  |
| 1. grapes | 1 cup |  |  |  |  |
| 1. Others: Banana, medium /Apple, fresh, small | 1 item |  |  |  |  |
| 1. Dried fruits: raisins (2 Tbsp), dates (2), apricots (4) | 1 serving |  |  |  |  |
| 1. Fresh fruit juice | 1 cup |  |  |  |  |
| 1. Fruit drinks: canned/bottled | 1 cup |  |  |  |  |
| **Vegetables** |  |  |  |  |  |
| 1. Salad – green: lettuce, celery, green peppers, cucumber | 1 cup |  |  |  |  |
| 1. Dark green or deep yellow vegetables (e.g.: spinach, hindbeh,, carrots , …) | 1 cup |  |  |  |  |
| 1. Tomatoes, fresh, medium | 1 item |  |  |  |  |
| 1. Corn / green peas, cooked | 1 cup |  |  |  |  |
| 1. potato, baked / boiled / mashed | 1 item |  |  |  |  |
| 1. Squash, summer (kussa), Eggplant /cooked | 1 cup |  |  |  |  |
| 1. Cauliflower/ Cabbage/ broccoli | 1 cup |  |  |  |  |
| **Meat & Alternates** | **Serving size** | **Day** | **Week** | **Month** | **Rarely / Never** |
| 1. Legumes: lentils, broad beans, chickpeas, etc., cooked | 1 cup |  |  |  |  |
| 1. Nuts and seeds: peanuts, almonds, sunflower seeds, etc. | 1 cup |  |  |  |  |
| 1. Red Meat | 1 item (3 oz.) |  |  |  |  |
| 1. Poultry | 1 item (3 oz.) |  |  |  |  |
| 1. Fish, ( including Tuna) | 1 serving (3 oz.) |  |  |  |  |
| 1. Eggs, whole, large | 1 item |  |  |  |  |
| 1. Organ Meats( Liver, kidneys, brain) | 1 cup |  |  |  |  |
| 1. Luncheon meats: Mortadell, Jambon, salami, turkey, etc. | 1 slice (20g) |  |  |  |  |
| 1. Sausages, makanek, hot dogs | 1 item (30g) |  |  |  |  |
| **Fats and oils** |  |  |  |  |  |
| 1. Oil: corn / sunflower / soy/olive | 1 Tbsp |  |  |  |  |
| 1. Olives | 1 item |  |  |  |  |
| 1. Butter/ghee | 1 Tbsp |  |  |  |  |
| 1. Mayonnaise | 1 Tbsp |  |  |  |  |
| **Sweets &Desserts** |  |  |  |  |  |
| 1. Cake, Cookies ,Donut, muffin, croissant | 1 item |  |  |  |  |
| 1. Ice cream | 1 cup |  |  |  |  |
| 1. Chocolate bar | 1 item |  |  |  |  |
| 1. Sugar, , honey, jam, molasses | 1 Tbsp |  |  |  |  |
| 1. Arabic sweets, baklawa, maamoul, Knefeh | 1 item (40g) |  |  |  |  |
| **Beverages** |  |  |  |  |  |
| 1. Soft drinks, regular (1 can = 1½ cup) | 1½ cup (11 fl. oz) |  |  |  |  |
| 1. Soft drinks, diet (1 can = 1½ cup) | 1½ cup (11 fl. oz) |  |  |  |  |
| 1. Turkish coffee (1 small cup = ¼ cup) | ¼ cup (2 fl oz) |  |  |  |  |
| 1. Coffee/Nescafe or tea | 1 cup |  |  |  |  |
| 1. Hot chocolate or cocoa | 1 cup |  |  |  |  |
| 1. Beer, regular (1 can = 1½ cup) | 1½ cup |  |  |  |  |
| 1. Wine: red, white, or blush | ½ cup (4 fl. oz) |  |  |  |  |
| 1. Liquor: whiskey, vodka, gin, rum | 1/6 cup (1.5 fl oz.) |  |  |  |  |
| **Miscellaneous** |  |  |  |  |  |
| 1. Manaeesh, zaatar, cheese | 1 large |  |  |  |  |
| 1. French fries | 1 cup |  |  |  |  |
| 1. Chips: potato, corn, tortilla | 1 cup |  |  |  |  |
| 1. Falafel sandwich, medium | 1 item |  |  |  |  |
| 1. Chawarma sandwich, medium | 1 item |  |  |  |  |
| 1. Burgers( Beef, chicken, fish) | 1 item |  |  |  |  |
| 1. Pizza | 1 slice |  |  |  |  |

**Are there any other foods not mentioned above that you usually eat at least once per week?**

| **Other foods that you usually eat at least once /week** | **Usual serving size** | **Servings/week** |
| --- | --- | --- |
|  |  |  |
|  |  |  |

**إنتهــــــت المقابلة**

**مع الشكر الجزيل**

**توقيع الباحث الميداني -------------------------**

**الجامعة الاميركية في بيروت**

**استبيان مسح سلوكيات وعوامل الخطورة**

**للأمراض غير السارية في لبنان– /092008**

**قياس الضغط، الطول والوزن ومحيط الخصر والورك**

| **Physical & Anthropometric Measurements** |
| --- |
| **step 1** |

| **Measurements** | | |  |
| --- | --- | --- | --- |
| **No. 2** | **No. 1** | **Blood Pressure** | **Code** |
|  |  | **Systolic** | BP_SYS (1& 2) |
|  |  | **Diastolic** | BP_DIAS (1& 2) |

| **Measurements** | | |  |
| --- | --- | --- | --- |
| **No. 2** | **No. 1** |  | **Code** |
|  |  | **Height (cm)** | HT (1& 2) |
|  |  | **Weight (kg)** | WT(1& 2) |
|  |  | **Waist circumference (cm)** | WAIST_CIRC (1& 2) |
|  |  | **Hip circumference (cm)** | HIP_CIRC (1& 2) |
|  | | | |
|  | | | |
| SKINFOLD | | | |
|  |  | **Triceps (mm)** | TRICEPS (1& 2) |
|  |  | **Biceps (mm)** | BICEPS (1& 2) |
|  |  | **Subscapular (mm)** | SUBSCAP (1& 2) |
|  |  | **Suprailiac (mm)** | SUPAILIAC (1& 2) |

**قياس السكري والدهنيات (فقط 18 سنة وما فوق)**

| **Biochemical Measurements**  **step2** |
| --- |

|  | **Date of blood test** | Date_blood |
| --- | --- | --- |
|  | **Fasting Blood Sugar (mg/dl)** | FBS |
|  | **Cholesterol (mg/dl)** | TOT_CHOL |
|  | **HDL (mg/dl)** | HDL |
|  | **LDL (mg/dl)** | LDL |
|  | **Triglyceride (mg/dl)** | TG |

**Name & Signature of Nurse/Dr : -----------------------------**
